# Correction to “European Network on Optimizing Treatment With Therapeutic Antibodies in Chronic Inflammatory Diseases: Overview, Progress and Perspectives”

**DOI:** 10.1111/cts.70696

**Published:** 2026-08-06

K. Tachkov , M. Rumano , J. Bluett , et al., “European Network on Optimizing Treatment With Therapeutic Antibodies in Chronic Inflammatory Diseases: Overview, Progress and Perspectives,” Clinical and Translational Science 19, no. 6 (2026): e70643, 10.1111/cts.70643.42321148 PMC13282160

In the article cited above, two changes have been made to the Reference list to ensure that the text citations align properly with the list.

(1) Reference 21 (G. Chauca Strand, C. Bonander, N. Jakobsson, N. Johansson, and M. Svensson, “Assessment of the Clinical and Cost‐Effectiveness Evidence in the Reimbursement Decisions of New Cancer Drugs,” *ESMO Open* 7, no. 6 (2022): 100569, https://doi.org/10.1016/j.esmoop.2022.100569) has been removed.

(2) G. Petrova et al. (formerly reference 26) has been moved after N. Holford and Z. Petric (formerly reference 27).

The corrected reference list, incorporating the changes outlined above, is shown below.

21 S. W. Syversen, G. L. Goll, K. K. Jorgensen, et al., “Effect of Therapeutic Drug Monitoring vs Standard Therapy During Infliximab Induction on Disease Remission in Patients With Chronic Immune‐Mediated Inflammatory Diseases: A Randomized Clinical Trial,” *Journal of the American Medical Association* 325, no. 17 (2021): 1744–1754.

22 B. Lewis and L. Sheiner, “Computer‐Aided Long‐Term Anticoagulation Therapy,” *Computers and Biomedical Research* 2, no. 6 (1969): 507–518, https://doi.org/10.1016/0010‐4809(69)90030‐5.

23 R. W. Jelliffe, J. Buell, and R. Kalaba, “Reduction of Digitalis Toxicity by Computer‐Assisted Glycoside Dosage Regimens,” *Annals of Internal Medicine* 77, no. 6 (1972): 891–906, https://doi.org/10.7326/0003‐4819‐77‐6‐891.

24 I. K. Minichmayr, E. Dreesen, M. Centanni, et al., “Model‐Informed Precision Dosing: State of the Art and Future Perspectives,” *Advanced Drug Delivery Reviews* 215 (2024): 115421, https://doi.org/10.1016/j.addr.2024.115421.

25 N. Holford and Z. Petric, “The Rational Basis for Personalized Treatment Using Concentration‐Guided Dosing,” *Therapeutic Drug Monitoring* 47 (2025): 1–8, https://doi.org/10.1097/FTD.0000000000001350.

26 G. Petrova, K. Tachkov, G. Kararigas, M. Itik, E. Drakalska, and D. Mulleman, “Statement on Education and Scientific Research in the Field of Therapeutic Drug Monitoring by the COST Action ENOTTA CA21147,” *Pharmacia* 73 (2026): 1–4, https://doi.org/10.3897/pharmacia.73.e184155.

We apologize for this error.

